# Identification of miRNAs Involved in Olfactory Regulation in Antennae of Beet Webworm, *Loxostege sticticalis* (Lepidoptera: Pyralidae)

**DOI:** 10.3390/life14121705

**Published:** 2024-12-23

**Authors:** Yu Zhang, Yanyan Li, Haibin Han, Xiaoling Wang, Shujing Gao, Qing Zhao, Halima Bieerdebieke, Linbo Xu, Qicong Zang, Hui Wang, Penghua Bai, Kejian Lin

**Affiliations:** 1 Key Laboratory of Biohazard Monitoring, Green Prevention and Control for Artificial Grassland, Ministry of Agriculture and Rural Affairs, Institute of Grassland Research of Chinese Academy of Agricultural Sciences, Hohhot 010010, China; zhangyu11@caas.cn (Y.Z.); gaoshujing@caas.cn (S.G.); zhaoqing01@caas.cn (Q.Z.); xulinbo@caas.cn (L.X.); wh1978xx@163.com (H.W.); 2 Research Center for Grassland Entomology, Inner Mongolia Agricultural University, Hohhot 010020, China; liyanyan@imau.edu.cn (Y.L.); hhb.25@163.com (H.H.); 3 Xilin Gol League Agricultural and Animal Husbandry Technology Promotion Center, Xilinhot 026000, China; xlwang_009492@163.com; 4 The Center for Grassland Biological Disaster Prevention of Xinjiang Uygur Autonomous Region, Urumqi 830049, China; arna_0305@163.com; 5 Heilongjiang Province Grassland Station, Harbin 150069, China; zqc0451@126.com; 6 Institute of Plant Protection, Tianjin Academy of Agricultural Sciences, Tianjin 300384, China

**Keywords:** *Loxostege sticticalis*, microRNAs, target prediction, olfactory-related genes, expression levels

## Abstract

The beet webworm, *Loxostege sticticalis*, is a typical migratory pest. Although miRNAs participate in many physiological functions, little is known about the functions of miRNAs in olfactory regulation. In this study, 1120 (869 known and 251 novel) miRNAs were identified in the antennae of *L. sticticalis* by using high-throughput sequencing technology. Among the known miRNAs, 189 from 49 families were insect-specific, indicating that these miRNAs might play unique roles in insects. Furthermore, based on the Gene Ontology (GO) and Kyoto Encyclopedia of Genes and Genomes (KEGG) analyses, we found that 3647 and 1393 miRNAs were associated with localization and the regulation of localization, respectively, and 80 miRNAs were enriched in the neuroactive ligand–receptor interaction pathway. These miRNAs might be involved in the olfactory system of *L. sticticalis*. Notably, qRT-PCR showed that most of the tested miRNAs presented similar expression patterns compared with the RNA-seq data and that *miR-87-3*, *novel-miR-78*, and *novel-miR-142* were significantly differentially expressed in the antennae of males and females. In addition, 21 miRNAs were predicted to target 23 olfactory genes, including 10 odorant-binding proteins (OBPs), 3 chemosensory proteins (CSPs), 4 odorant receptors (ORs), 1 ionotropic receptor (IR), and 5 gustatory receptors (GRs). The olfactory-related miRNAs exhibited low-abundance transcripts, except *undef-miR-55* and *undef-miR-523*, and gender-biased expression was not observed for olfactory-related miRNAs. Our findings provide an overview of the potential miRNAs involved in olfactory regulation, which may provide important information on the function of miRNAs in the insect olfactory system.

## 1. Introduction

Small regulatory non-coding RNA molecules (approximately 22 nucleotides in length), known as microRNAs (miRNAs), have been proven to be key regulators at the post-transcriptional level. miRNAs bind to the 3′-untranslated regions (3′-UTRs) of target genes involved in various biological processes to regulate their expression [1,2,3]. Since the first miRNAs (*lin-4* and *let-7*) were discovered in *Caenorhabditis elegans* [1], increasing numbers of miRNA genes have been utilized and identified in vertebrates, plants, arthropods, and viruses via molecular cloning methods, computational approaches, and high-throughput sequencing [4,5,6]. Researches on miRNAs in insects has mainly focused on model insects, such as *Drosophila melanogaster* [7,8,9,10,11], *Anopheles gambiae* [12], *Aedes aegypti* [13,14], *Apis mellifera* [15], and *Bombyx mori* [16]. Numerous miRNAs have been identified in multiple insect species, which contributed to many special biological processes, including development, reproduction, and behavior [17,18,19,20,21,22]. In *Drosophila*, researchers have found that miRNAs could regulate behavioral effects, suggesting that miRNAs may be core components of the genetic programs underlying behavioral control in other insects [23]. As predicted, in locusts, *miRNA-133* was found to be involved in behavioral aggregation and controlling dopamine synthesis [24]. According to the above studies, researchers speculated that the miRNA regulation mechanism of the insect olfactory system might be a common phenomenon in many kinds of insects [4,5]. This speculation was initially verified in *Drosophila*. The transcription factor Nerfin-1 was found to be down-regulated by *miR-279*, which leaded to the formation of CO_2_-sensing neurons in the maxillary palps [25]. Another miRNA, *miR-276a*, is required in mushroom body neurons for the formation of memory and in the ellipsoid body for naive responses to odors in *Drosophila*, highlighting the importance of miRNA-mediated gene regulation for behavioral responses [26]. In addition, the Ataxin-2 protein was found to be required for miRNA function for long-term olfactory habituation in *Drosophila* [27]. In addition to *Drosophila*, there is increasing evidence showing that miRNAs are involved in other insects’ chemical communication. For example, *miRNA-9a* was identified to be associated with locust olfactory attraction after the activation and inhibition of Dopamine receptor 1 [28].

The antennae are the most important olfaction organs in insects and involved in multiple behaviors, such as feeding, mating, and oviposition [29,30,31,32,33]. The insect olfactory system is highly complicated and sensitive, and multiple olfactory genes play active roles in odorant detection, including odorant-binding receptors (OBPs), chemosensory proteins (CSPs), odorant receptors (ORs), ionotropic receptors (IRs), gustatory receptors (GRs), odorant-degrading enzymes (ODEs), and sensory neuron membrane proteins (SNMPs) [34,35,36]. In recent years, studies have discovered that miRNAs were potentially involved in olfactory regulation [17,19,37,38]. In *Apolygus lucorum*, 15 miRNAs were predicted to target 16 olfactory genes [19]. In *Microplitis mediator*, 17 miRNAs were highly expressed in the antennae and were predicted to be associated with olfactory genes, including OBPs, ORs, and IRs [17]. In *Holotrichia parallela*, 13 miRNAs were successfully shown to participate in olfactory regulation [37]. Consequently, the above studies provide solid evidence that miRNAs participate in insect behavior by regulating the olfactory system.

The beet webworm, *Loxostege sticticalis* Linnaeus (Lepidoptera: Pyralidae), a typical migratory pest, is one of the National Class I list of crop insect pests and mainly occurs in northern China [39]. Based on the transcriptome database, multiple olfactory-associated genes have been identified in *L. sticticalis*, including 34 OBPs, 10 CSPs, 54 ORs, 18 IRs, 13 GRs, and 2 SNMPs [40], and the functions of some olfactory-associated genes have been verified [39,41,42,43,44]. For example, LstiPR2 is a pheromone receptor of *L. sticticali* and showed responded to the major sex pheromone compound (*E*11-14:OAc) specifically [39]. However, limited information is available about the functions of miRNAs in olfactory regulation in this organism. Based on the above statement, we hypothesize that there must be some miRNAs regulating the olfactory genes in this species. Our aims were to identify the olfactory-related miRNAs in *L. sticticalis* and predict their targets.

## 2. Materials and Methods

### 2.1. Insect Rearing and Tissue Collection

*L. sticticalis* larvae were collected from Dalad Banner, Ordos, Inner Mongolia, China (40°18′49″ N, 109°55′50″ E). Briefly, the larvae were reared with fresh *Chenopodium album* under the following conditions: temperature, 22 ± 1 °C; relative humidity, 75 ± 5%; and photoperiod, 16:8 (L:D). The last-instar larvae were transferred in a box with approximately 15% humidity and sandy soil until pupation. Newly emerged adults were fed 5% honey solution. Three-day-old male and female adults’ antennae were dissected, immediately put into liquid nitrogen, and stored at −80 °C for high-throughput sequencing and expression profiling analyses.

### 2.2. RNA Isolation and Small RNA Library Construction

The antennae were dissected from 40 three-day-old male and female adults, and total RNA was extracted by using TRIzol^®^ Reagent (Invitrogen, Carlsbad, CA, USA), following the manufacturer’s instructions. The concentration, quality, purity, and integrity of the total RNA were determined with a NanoDrop NC2000 (Thermo Scientific, Waltham, MA, USA) and an Agilent 2100 Bioanalyzer (Agilent Technologies, Waldbronn, Germany). A small RNA (sRNA) library was constructed by using the NEBNext Multiplex Small RNA Library Prep Set for Illumina (New England Biolabs, Ipswich, Suffolk, GBR) according to the manufacturer’s instructions. In brief, 1 μg of total RNA from *L. sticticalis* antennal samples was ligated to a 3′ adapter and a 5′ adapter by using Ligation Enzyme Mix; the resulting sample was taken as the template for reverse transcription, which was performed with Superscript II reverse transcriptase. Subsequently, fragments of 300 bp to 400 bp in length were selected and enriched through PCR amplification according to the manufacturer’s protocols. Small RNA libraries were analyzed for QC, and the average size of the inserts was determined to be approximately 140 bp to 150 bp. The sequencing library was quantified by using an Agilent high-sensitivity DNA assay on a Bioanalyzer 2100 system (Agilent Technologies, Waldbronn, Germany) and was then sequenced on the NovaSeq 6000 platform (Illumina, San Diego, CA, USA) at Shanghai Personal Biotechnology Cp. Ltd. (Personalbio, Shanghai, China).

### 2.3. Bioinformatic Analysis

The quality of the raw data was calculated; then, the data were filtered by using Personalbio company’s self-developed script to remove the 3′ adapters and low-quality sequences and obtain clean data. The clean reads from 18 nt to 36 nt in length were filtered, and deduplication was performed to obtain unique reads for subsequent analysis. The genome of *L. sticticalis* was used as the reference (BioProject, PRJNA1118492); the unique reads were compared with the Rfam databases [45] by using Blast; then, the reads were annotated and other non-coding RNAs, including transfer RNA (tRNA), ribosomal RNA (rRNA), small nuclear RNA (snRNA), and small nucleolar RNA (snoRNA), were discarded. The unique reads remaining were blasted in the miRBase22 database (http://www.mirbase.org/, accessed on 10 November 2023) [46] to identify known miRNAs. For sequences that were not annotated with any information, we used mireap (v0.2) for new-miRNA prediction analysis and RNAfold to map the secondary structure. Furthermore, Pearson correlation coefficients were used to assess the reliability of the transcript measurements of the six constructed libraries and biological replicates. For the known miRNAs identified here, the mature miRNA sequences of closely related species in miRbase (release 23.10) were aligned with Blast with the aim of achieving conservation across species.

### 2.4. Expression Level of miRNA Analysis Based on Transcripts and qRT-PCR

The read count value of the miRNAs was calculated based on the number of sequences aligned to the mature miRNAs. The first abundance value in the miRNAs with the same name was chosen for subsequent analysis. DESeq (v1.18.0) was employed to analyze the differentially expressed miRNAs, as indicated by transcripts with |log2FoldChange|>1 and *p*-value < 0.05. Furthermore, quantitative real-time PCR (qRT-PCR) was conducted to compare the transcription levels of the ten most abundant known and novel miRNAs of male and female *L. sticticalis* antennae. The antennae were collected from three-day-old male and female *L. sticticalis* adults, and total RNA was isolated by using a Quick-RNATM Kit (Genstone Biotech Co., Ltd., Beijing, China). Quality was checked with 1% agarose gel electrophoresis (AGE), and the purity and concentration of the RNA were tested by using a NanoDrop 2000 (Thermo Fisher Scientific, Wilmington, DE, USA). First-strand cDNA was synthesized with a Mir-XTM miRNA First-Strand Synthesis Kit (TaKaRa, Dalian, China) according to the manufacturer’s instructions. The assay of quantitative real-time reverse transcription PCR (qRT-PCR) was performed by using the Hieff^@^ qPCR SYBR Green Master Mix (Low Rox Plus) (Yeasen Biotech Co., Ltd., Shanghai, China) and QuantStudio 5 (Thermo Fisher Scientific, Wilmington, DE, USA). Specific primers (Table S1) for the quantification of the target genes were designed by using the online website Primer 3.0 (http://primer3.ut.ee/, accessed on 19 August 2024). The qRT-PCR reaction mixture had a total volume of 20 μL and included 10 μL of Hieff^@^ qPCR SYBR Green Master Mix (Low Rox Plus), 1 μL of template cDNA, 0.4 μL (10 μmol L^−1^) of primers (sense and antisense), and 8.2 μL of RNase-free H_2_O. The qRT-PCR reaction conditions were as follows: 95 °C for 5 min; then, 40 cycles at 95 °C for 10 s, 55 °C for 20 s, 72 °C for 20 s; and melt curve stages at 95 °C for 15 s, 60 °C for 1 min, and 95 °C for 15 s. The qRT-PCR tests included three biological replicates, each with three technical repeats. Relative expression was calculated by using the 2^−∆∆CT^ method with U6 snRNA as the reference gene [47].

### 2.5. Differentially Expressed miRNA Enrichment Analysis

Gene Ontology (GO; http://geneontology.org/, accessed on 10 November 2023) and Kyoto Encyclopedia of Genes and Genomes (KEGG; http://www.kegg.jp/, accessed on 10 November 2024) enrichment analyses were performed on the target genes of the differentially expressed miRNAs. We used topGO (v2.50.0) to perform GO enrichment analysis on the target genes of the differential miRNAs; first, we calculated the *p*-value with the hypergeometric distribution method (the standard of significant enrichment is a *p*-value < 0.05); then, we determined the GO terms with significantly enriched differentially expressed genes to determine the main biological functions performed by the differentially expressed genes. We further used clusterProfiler (v4.6.0) software to carry out the enrichment analysis of the KEGG pathways of the target genes of the differential miRNAs, focusing on significant enrichment pathways with *p*-values < 0.05.

### 2.6. Chemosensory-Related Target Gene Prediction and Expression Levels Based on Transcript Analysis

The antennal transcriptome data of *L. sticticalis* formed the candidate target gene local library and included 34 odorant-binding proteins (OBPs), 10 chemosensory proteins (CSPs), 54 odorant receptors (ORs), 18 ionotropic receptors (IRs), 13 gustatory receptors (GRs), and 2 sensory neuron membrane proteins (SNMPs) [40]. The putative targets of miRNAs (known and novel) identified from the antennae of *L. sticticalis* were determined with miRanda (v3.3a) [48] and RNAhybrid (7.0) [49]. The cut-offs of the two computational prediction algorithms were a score ≥ 140 and minimum free energy (MEF) ≤ −25 Kcal/mol for miRanda, and MEF ≤ −25 Kcal/mol and *p*-value ≤ 0.05 for RNAhybrid. The targets marked by both algorithms were chosen as the predicted targets. Moreover, the FPKM values of small RNA sequencing were used to analyze the sex differences in the antennae of *L. sticticalis*.

### 2.7. Data Analysis

The data were analyzed by using SPSS 17.0 (IBM Inc., Chicago, IL, USA), and graphs were created by using GraphPad Prism 7.0 (GraphPad Software Inc., CA, USA). The olfactory-related miRNA expression levels in both sexes were analyzed by using *t*-tests (*p* < 0.05) (IBM, Endicott, NY, USA). Differences in the expression of antenna-biased miRNAs between males and females were analyzed with Student’s *t*-test with SPSS Statistics, version 17 (SPSS Inc., Chicago, IL, USA).

## 3. Results

### 3.1. Overview of Small RNA Sequencing Data

Male and female antennae of *L. sticticalis* were used to perform small RNA sequencing to identify miRNAs. In total, 87,477,121.00 (≥13.4 million per library) raw data were generated, and after removing 3′ adaptor sequences and low-quality reads, the remaining reads ranging from 18 to 36 nt were kept (Figure S1). A total of 104,348,742 reads were retained and mapped to the reference genome of *L. sticticalis* (unpublished). Three libraries for male antennae (12,662,289.00, 10,290,098.00, and 12,543,503.00) and three for female antennae (12,156,083.00, 11,349,143.00, and 13,119,518.00) were obtained (Table 1). The clean reads were aligned with the databases Rfam and miRbase and divided into four categories, including rRNAs (1.18%), tRNAs (1.95%), snRNAs (2.08%), and snoRNAs (15.46%) (Table 1, Figure S2), and the remaining reads were used to predict known and novel miRNAs. Furthermore, the analysis of the relativity between any two libraries showed that the Pearson correlation coefficients ranged from 0.91 to 1 (Figure S3).

### 3.2. Identification and Analysis of miRNAs

**Figure 1:**
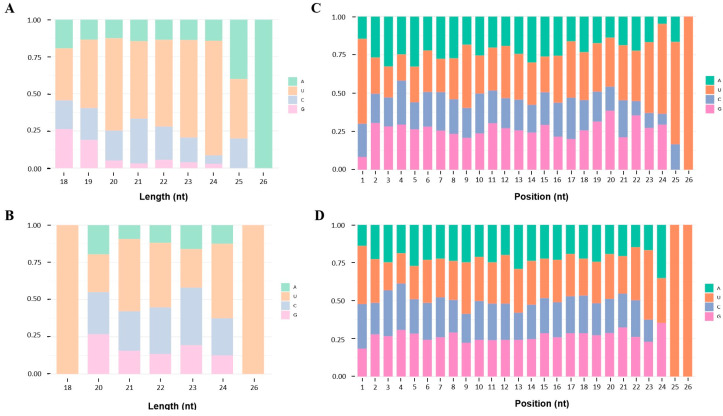
The frequencies of the first and the other bases of the miRNAs. (**A**,**B**) The first nucleotide base of the known and novel miRNAs, respectively. The *X*-axis represents the length of different miRNAs, and the *Y*-axis represents the percentage of frequency of a four-base distribution of miRNAs of different lengths. (**C**,**D**) The nucleotide bases of known and novel miRNAs at each position. The *X*-axis represents the bases of the miRNAs from 1 to 26, and the *Y*-axis represents the percentage of different base distributions at each position of the miRNAs.

**Figure 2:**
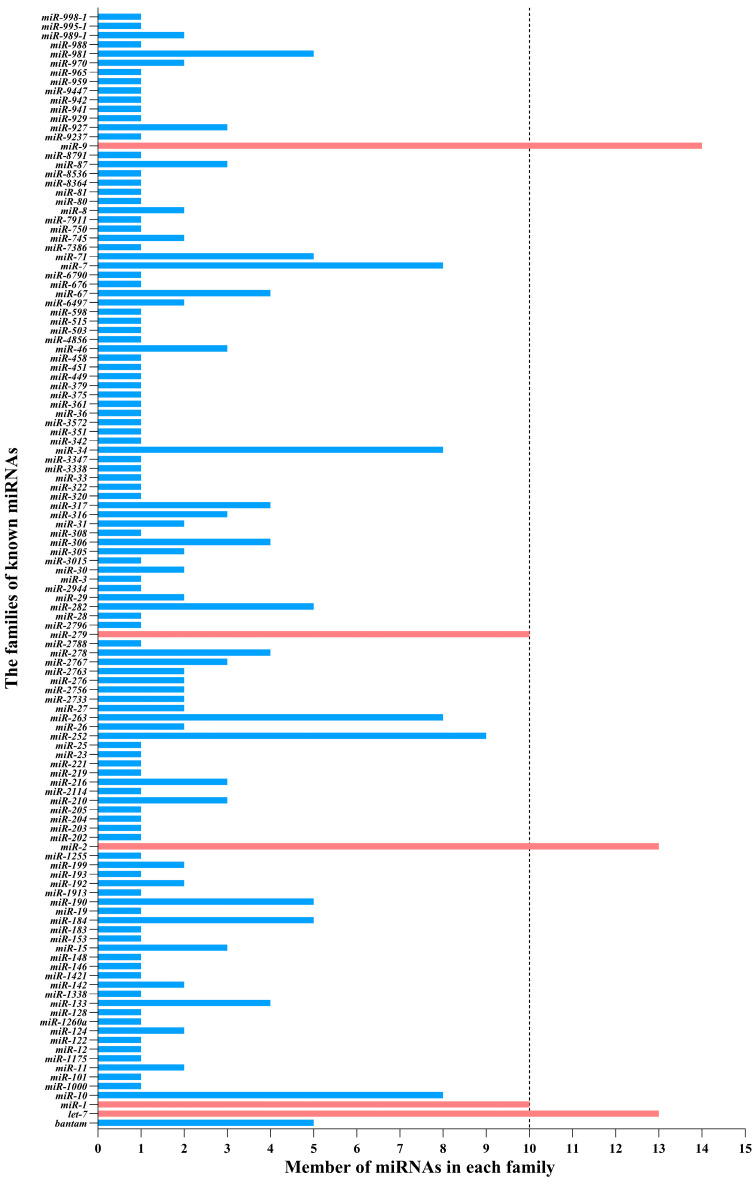
The members of known miRNAs identified in *Loxostege sticticalis* antennae in each of the miRNA families. The families with more than ten members are marked in red color, while the others are shown in blue color.

The bioinformatic analysis showed that 1120 miRNAs were identified (Table S2), including 869 unique known miRNAs, according to the homology miRNAs in miRBase, and 251 novel miRNAs, according to the precursor sequences and the estimation of their randfold value (Table 1). The identified miRNAs ranged from 21 to 23 nt in length, with a characteristic peak at 22 nt (Figure S1). Both known and novel miRNAs showed a bias to U (uridine) in the first position of the sequence, and the base frequency of each position in all the miRNA reads showed that U and A (adenine) occurred more frequently than C (cytosine) and G (guanine) (Figure 1). In addition, 254 known miRNAs were categorized into 121 families, according to the conservation of the sequences across different species; however, the family affinity of the remaining 582 miRNAs could not be categorized. Furthermore, ten families which had relatively more members than the other sets were discovered. Specifically, the *miR-9* family includes 14 of the identified miRNAs, the *let-7* and *miR-2* families include 13 of the identified miRNAs, the *miR-1* and *miR-279* families include 10 of the identified miRNAs, and the *miR-252* family includes 9 of the identified miRNAs. Four families (*miR-10*, *miR-263*, *miR-34*, and *miR-7*) include eight of the identified miRNAs, and six families (*bantam*, *miR-184*, *miR-190*, *miR-282*, *miR-71*, and *miR-981*) include five of the identified miRNAs. Apart from the above families, the number of each of the remaining families was less than five (Figure 2). The conservative analysis showed that one of the identified families (*miR-67*) belongs to the most conserved category and is present in arthropods, nematodes, and vertebrates. Six of the identified families (*miR-1*, *miR-10*, *miR-11*, *miR-1175*, *miR-7*, and *miR-9*) belong to the highly conserved category and are present in arthropods and vertebrates. Three of the identified families (*bantam*, *miR-34*, and *miR-71*) belong to the invertebrate-specific category and are present in arthropods and nematodes. Four of the identified families (*miR-252*, *miR-263*, *miR-279*, and *miR-317*) are only found in arthropods, and two identified families (*miR-80* and *miR-81*) are only found in nematodes. A total of 49 identified families (189 miRNAs) fall into the insect-specific category and include *let-7*, *bantam*, *miR-1*, *miR-10*, *miR-11*, *miR-1000*, *miR-1175*, *miR-12*, *miR-184*, *miR-190*, *miR-2*, *miR-210*, *miR-216*, *miR-25*, *miR-252*, *miR-263*, *miR-276*, *miR-279*, *miR-2763*, *miR-2767*, *miR-278*, *miR-2788*, *miR-2796*, *miR-28*, *miR-2944*, *miR-34*, *miR-305*, *miR-306*, *miR-308*, *miR-31*, *miR-316*, *miR-317*, *miR-33*, *miR-3338*, *miR-46*, *miR-67*, *miR-7*, *miR-71*, *miR-745*, *miR-750*, *miR-87*, *miR-9*, *miR-927*, *miR-929*, *miR-970*, *miR-981*, *miR-988*, *miR-989*, and *miR-998*. A total of 26 of the identified families fall into the vertebrate-specific category and include *miR-965*, *miR-101*, *miR-122*, *miR-124*, *miR-128*, *miR-133*, *miR-142*, *miR-146*, *miR-15*, *miR-19*, *miR-192*, *miR-193*, *miR-199*, *miR-202*, *miR-23*, *miR-27*, *miR-29*, *miR-30*, *miR-322*, *miR-351*, *miR-361*, *miR-458*, *miR-515*, *miR-598*, *miR-941*, and *miR-942*. *L. sticticalis* shares 42 conserved families with *B. mori*, and the number of miRNA-conserved families in insects is larger than that in arthropods. Notably, 12 of the identified families (*miR-1338*, *miR-148*, *miR-183*, *miR-26*, *miR-2733*, *miR-320*, *miR-342*, *miR-379*, *miR-451*, *miR-6497*, *miR-8*, and *miR-8536*) were found not to belong to any category, which may indicate that they are species-specific in *L. sticticalis*. The details of the conservative analysis are listed in Table S3.

### 3.3. Abundance of miRNAs in Antennae of Loxostege sticticalis

**Figure 3:**
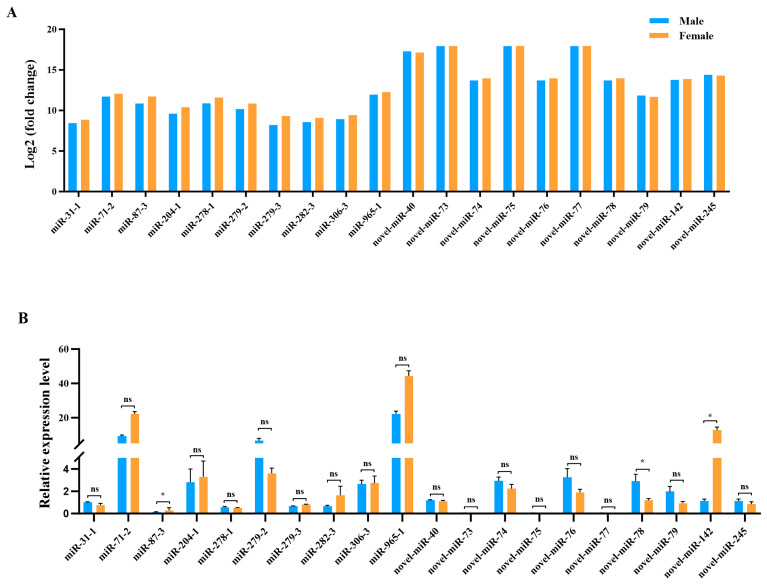
The ten most abundant known and novel miRNA expression levels in male and female antennae of *Loxostege sticticalis*. (**A**,**B**) The expression levels of the transcripts and qRT-PCR, respectively. Fold change: normalization values in small RNA sequencing data. “ns” indicates no significant differences and “*” significant differences at the *p* < 0.05 level.

Firstly, the expression levels of the miRNAs were assessed with the counts per million (CPM) formula. The most abundant known miRNA was *miR-965-1*, followed by *miR-71-2*, *miR-87-3*, *miR-278-1*, and *miR-279-2*. Moreover, the most highly expressed novel miRNA was *novel-miR-73*, followed by *novel-miR-75*, *novel-miR-77*, and *novel-miR-40*. Notably, the average expression levels of the novel miRNAs were significantly higher than those of the known miRNAs, as shown in Table 2. Details on the abundance of the remaining known and novel miRNAs are listed in Table S2. To verify the RNA-Seq results, qRT-PCR analysis was performed to evaluate the expression of the 20 most abundant miRNAs, and the results show that the majority of the tested genes presented similar expression patterns compared with the RNA-Seq data. For example, *miR-965-1* exhibited the highest expression among the known miRNAs according to both RNA-Seq and qRT-PCR (Figure 3). Notably, three miRNAs, i.e., *miR-87-3* (*p* = 0.027), *novel-miR-78* (*p* = 0.033), and *novel-miR-142* (*p* = 0.022), had higher expression levels in female than in male antennae. However, the other miRNAs did not have significant sex-specific expression differences between female and male antennae.

### 3.4. Differentially Expressed miRNAs in Antennae of Loxostege sticticalis

**Figure 4:**
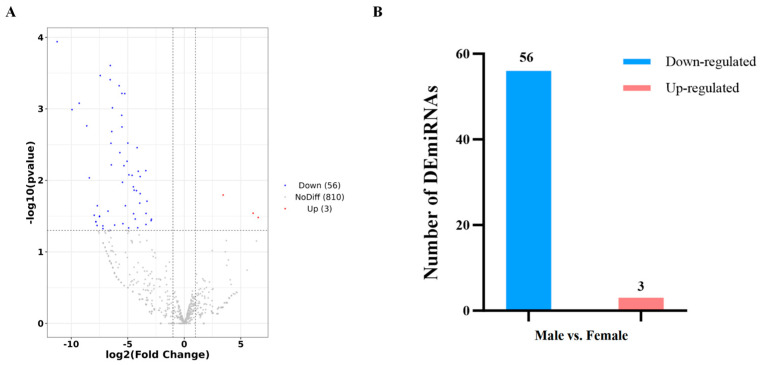
Analysis results of differentially expressed miRNAs (DEmiRNAs) in the antennae of *Loxostege sticticalis*. (**A**) Volcano plot of DEmiRNAs in male vs. female libraries. (**B**) Number of DEmiRNAs in male and female antennae of *L. sticticalis*. Blue, red, and gray dots represent down-regulation, up-regulation, and no differences in expression levels, respectively.

Differentially expressed miRNAs in the male and female antennae of *L. sticticalis* were compared according to the criteria of *p*-value < 0.05 and log2 fold change ≤ 1. Notably, no novel miRNAs were significantly differentially expressed between the two sexes. However, it is noteworthy that 59 known miRNAs were significantly differentially expressed between the sexes, including 56 down-regulated miRNAs and 3 up-regulated miRNAs (Figure 4). Namely, the latter were *undef-miR-117*, *let-7-12*, and *undef-miR-235*, with log2 fold-change values of 6.543, 6.095, and 3.443, respectively. The most down-regulated miRNAs were *undef-miR-302*, *undef-miR-294*, and *undef-miR-361*, with log2 fold-change values of −11.248, −9.931, and −9.293, respectively (Table S4).

### 3.5. GO Functional Analysis and KEGG Pathway Enrichment of DEmiRNAs

**Figure 5:**
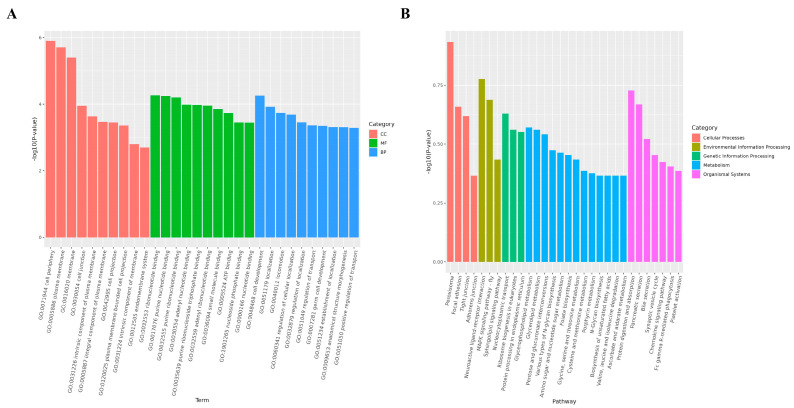
GO function analysis and KEGG pathway enrichment of DEmiRNAs. (**A**) Top 10 GO terms of each category of DEmiRNAs. *X*-axis represents term of GO level 2. *Y*-axis represents -log10 (*p*-value) enrichment of each term. (**B**) Top 30 KEGG pathways for DEmiRNAs. *X*-axis represents name of pathway. *Y*-axis represents -log10 (*p*-value) enrichment of each pathway.

The GO functional analysis showed that the predicted target genes were enriched in 19,882 terms in the three categories of cellular components (CCs), molecular functions (MFs), and biological processes (BPs) (Figure 5A and Figure S4A, Table S5). A total of 2392, 2157, and 4015 miRNAs were enriched in the terms cell periphery (GO:0071944), plasma membrane (GO:0005886), and membrane (GO:0016020) in the category of CCs, respectively. The MF terms were related to binding, with more than 1000 miRNAs being enriched in ribonucleotide binding (GO:0032553), purine nucleotide binding (GO:0017076), and purine ribonucleotide binding (GO:0032555). Regarding the terms in the BP category, 2044, 3647, and 1393 miRNAs were enriched in cell development (GO:0048468), localization (GO:0051179), regulation of localization (GO:0060341), and regulation of transport (GO:0032879). Furthermore, the results of the KEGG analysis show 253 functional pathways in the five categories of cellular processes, environmental information processing, genetic information processing, metabolism, and organismal systems, with 96, 80, 65, 59, and 75 miRNAs being enriched in peroxisome (ko04146), neuroactive ligand–receptor interaction (ko04080), nucleocytoplasmic transport (ko03013), glycerophospholipid metabolism (ko00564), and protein digestion and absorption (ko04974), respectively (Figure 5B and Figure S4B, Table S6).

### 3.6. Prediction of Chemosensory-Related Target Genes and Analysis of Transcript Abundance

**Figure 6:**
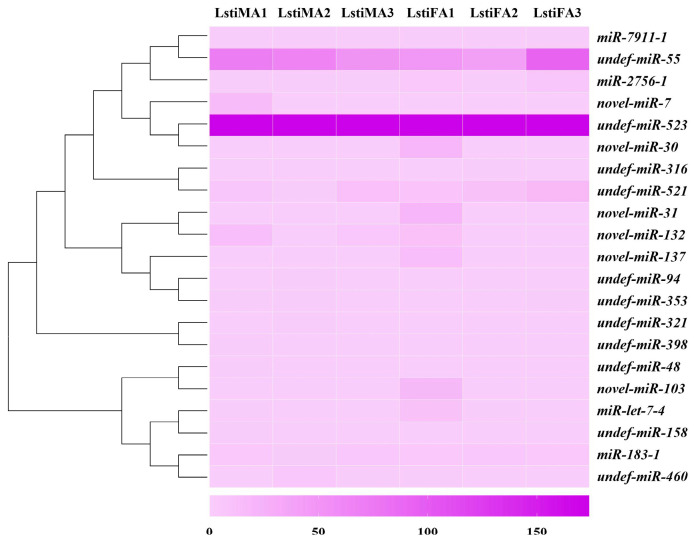
The heatmap analysis of chemosensory-related miRNAs in the antennae of males and females by transcript abundance in *Loxostege sticticalis*. MA, male antenna; FA, female antenna. The number (1–3) after each sample represents the biological replicate.

The software applications miRanda and RNAhybrid were used to screen the target genes for predicting olfactory-related miRNAs. By combining the two algorithms, 1120 targets were predicted for the known and novel miRNAs. Particularly, 21 unique miRNAs were found to target 23 unique olfactory-related genes in *L. sticticalis* (Table 3), including 10 OBPs (*LstiOBP4*, *LstiOBP10*, *LstiOBP12*, *LstiOBP13*, *LstiOBP15*, *LstiOBP17*, *LstiOBP22*, *LstiOBP26*, *LstiOBP29*, and *LstiPBP2*), 3 CSPs (*LstiCSP3*, *LstiCSP5*, and *LstiCSP10*), 4 ORs (*LstiOR3*, *LstiOR8*, *LstiOR43*, and *LstiOR48*), 1 IR (*LstiIR7g*), and 5 GRs (*LstiGR5b*, *LstiGR21b*, *LstiGR45*, *LstiGR63a*, and *LstiGR63a.2*). Some olfactory genes were targeted by the same miRNA; for instance, *LstiOBP26* and *LstiOR48* were targeted by *novel-miR-30*, and *LstiCSP10* and *LstiOR48* were targeted by *undef-miR-316*. Moreover, the transcript abundance of the chemosensory-related miRNAs was analyzed. Except for *undef-miR-55* and *undef-miR-523*, nineteen out of twenty-one miRNAs showed low expression levels and no gender bias based on the FPKM values (Figure 6).

## 4. Discussion

In recent years, miRNAs, a class of endogenous non-coding RNAs, have been found to regulate gene expression at the post-transcriptional level through cleavage or translation repression [2,3]. With the development of miRNA identification platforms, an increasing number of miRNAs have been characterized in multiple insect species, such as *M. mediator* [17], *A. lucorum* [19], and *Galeruca daurica* [50]. miRNAs have emerged as key gene regulators in diverse biological pathways in insect immunity [51], insecticide resistance [52,53], diapause [50], and development and behavior [21]. In recent years, some studies have shown that miRNAs play a crucial role in insect olfaction [17,19,37]. However, research on the regulation of miRNA functions has mainly focused on model insects, such as *Drosophila* [9,23,51], mosquito [13,14], and honeybee [15,54]. *L. sticticalis* is on the National Class I list of crop insect pests, and 34 OBPs, 10 CSPs, 54 ORs, 18 IRs, 13 GRs, and 2 SNMPs have been identified in *L. sticticalis* [40]. More importantly, the functions of some olfactory-associated genes have been verified [39,41,42,43,44]; for instance, LstiPR2 responds specifically to the major sex pheromone compound *E*11-14:OAc, which results in the activation of the “a” neuron in sensilla trichodea [39]. However, limited information about the functions of miRNAs in olfactory regulation in *L. sticticalis* is available.

In this study, sRNA libraries from the antennae of *L. sticticalis* males and females were constructed according to the genome data of this species. Moreover, the analysis of the Pearson correlation coefficients, which were higher than 0.91 between any two libraries, indicates the reliability of the transcript measurements among all constructed sRNA libraries [19]. In total, we identified 1120 miRNAs (869 known and 251 novel miRNAs) 21–23 nt in length, with a peak at 22 nt, which is characteristic of animal small RNAs [2,17,18]. The number of identified miRNAs in *L. sticticalis* was more than that in other species; for example, 99 miRNAs (76 known and 23 novel miRNAs) were identified in *H. parallela* [37], and 342 miRNAs (296 known and 46 novel miRNAs) were identified in *M. mediator* [17]. Additionally, this phenomenon may be due to the reference genome used for miRNA identification. In our study, the genome of *L. sticticalis* was employed as the reference to assemble and annotate the miRNAs. However, the miRNAs in *H. parallela* and *M. mediator* were identified by taking closely related species as the reference genomes, which led to a low number of miRNAs. Notably, 254 of the known miRNAs were categorized into 121 families, 50 of which are present across invertebrates and vertebrates, indicating a conserved function in evolution [17]. In addition, 36 families of insect-specific miRNAs were identified in *L. sticticalis*, suggesting that these miRNAs may play unique roles in insects. For example, in *A. aegypti*, *miR-277* belongs to an insect-specific miRNA family (*miR-277*), which controls lipid metabolism and reproduction by targeting insulin-like peptides 7 and 8 [14]. Three insect-specific miRNAs (*miR-932*, *miR-34-5p*, and *miR-279a*) were identified to play essential roles in early embryogenesis, memory, and foraging [54,55,56]. Our data pave the way to better understand the roles of miRNAs in many physiological functions, including insect reproduction, behavior, and olfactory regulation.

Among the identified miRNAs, *miR-965-1*, *miR-71-2*, *miR-87-3*, *miR-278-1*, and *miR-279-2* were listed as the most abundant in our sRNA libraries. Previously, it was found that *miR-279*, a member of the miR-279 family, was also listed as an abundantly expressed miRNA in *Plutella xylostella* larvae and was predicted to regulate immunity-related genes [57]. In *Drosophila*, researchers found that the miR-279 family plays an important role in the formation of both CO_2_ sensory neurons and memory [25,26]. Moreover, the most highly expressed novel miRNAs were *novel-miR-73*, *novel-miR-75*, *novel-miR-77*, and *novel-miR-40*. To verify the RNA-Seq results, qRT-PCR analyses were performed, and the results show that the majority of the tested genes presented similar expression patterns compared with the RNA-Seq data, suggesting that the latter were highly reliable [49]. Interestingly, three miRNAs, i.e., *miR-87-3*, *novel-miR-78*, and *novel-miR-142*, had higher expression levels in female than in male antennae, which indicates that they may participate in the process of sexual differentiation or gender-biased functions in *L. sticticalis*, such as locating oviposition sites [2,17,37,58].

Recently, an increasing number of studies have shown the potential involvement of miRNAs in olfactory regulation [17,19,37,38]. For example, *miR-9a-5p* was reported to target the olfactory gene *MmedOR18*, and *miR-7-5p* was predicted to target *MmedIR21a* in *M. mediator* [17]. In this study, a total of 21 miRNAs in the antennae of *L. sticticalis* were predicted to target 23 olfactory-related genes, including OBPs, CSPs, ORs, and GRs. Among them, four general odorant receptors (*LstiOR3*, *LstiOR8*, *LstiOR43*, and *LstiOR48*) were regulated by miRNAs and may be associated with the host–plant recognition process in *L. sticticalis* [30,31,43]. Furthermore, these miRNAs did not show different expression between male and female antennae, which indicates that they regulate non-gender-biased functions in the olfaction process, such as feeding and localization [30,43,59]. Similarly, in other insect species, miRNAs have been predicted to be involved in chemoreception through the regulation of the expression of olfactory genes. For example, in the beetle *H. parallela*, 13 miRNAs in the antennae have possible functions in the regulation of olfactory-associated genes, including OBPs and SNMPs [37]. In the parasitoid wasp *M. mediator*, 33 miRNAs could target 30 chemosensory genes, such as OBPs, CSPs, ORs, IRs, and GRs [17]. However, many research studies on miRNAs mainly remain in the stage of gene identification. Thus, it is necessary to strengthen the study on the function of miRNAs specific to olfactory regulation. Our findings provide a comprehensive overview of the miRNAs of *L. sticticalis* antennae and necessary valuable molecular information for future investigation, for instance, target gene prediction, function verification, and behavioral assays.

## 5. Conclusions

In conclusion, we identified 1120 (869 known and 251 novel) miRNAs in the antennae of *L. sticticalis*, 21 of which were predicted to target 23 olfactory-related genes, including OBPs, CSPs, ORs, IRs, and GRs. ORs play a critical role in the perception of chemical cues, and in our study, we found four *LstiORs* that are predicted to be regulated by miRNAs and which may be associated with host–plant recognition in *L. sticticalis*. Further studies should focus on the mechanisms of OR targeting in miRNAs, the regulation of behavior responses, and designing a potential strategy for controlling *L. sticticalis* through olfactory disruption.

## Figures and Tables

**Table 1:** sRNA libraries for male and female antennae of *Loxostege sticticalis*.

Group of Reads *	Number of Reads						Total Reads
	LstiMA-1	LstiMA-2	LstiMA-3	LstiFA-1	LstiFA-2	LstiFA-3	
Raw reads	14,333,482	15,869,882	14,117,311	13,781,722	13,420,963	15,953,761	87,477,121
Clean reads	12,662,289	10,290,098	12,543,503	12,156,083	11,349,143	13,119,518	72,120,634
Unannotated reads	783,277	216,504	742,613	462,210	339,366	717,068	3,261,038
rRNA	172,911	187,451	132,289	125,477	100,691	131,774	850,593
tRNA	14,261	13,209	11,501	8595	6247	9889	63,702
snRNA	3582	2602	3458	2337	1972	3714	17,665
snoRNA	2015	935	2213	1151	1047	2487	9848
Repeat	637	228	616	455	331	627	2257
Mapped reads	12,662,289	10,290,098	12,543,503	12,156,083	35,443,662	21,253,107	104,348,742
Known miRNA	471	415	508	433	422	450	869
Novel miRNA	71	28	67	66	67	77	251

* rRNA, ribosomal RNA; tRNA, transfer RNA; snRNA, small nuclear RNA; snoRNA, small nucleolar RNA.

**Table 2:** The ten most abundant known and novel miRNAs in male and female antennae of *Loxostege sticticalis*.

miRNA Name	Sequence (5′-3′)	Length (nt)	Expression Level (CPM *)
*miR-965-1*	TAAGCGTATAGCTTTTCCCATT	22	4450
*miR-71-2*	TGAAAGACATGGGTAGTGAGATT	23	3816
*miR-87-3*	GTGAGCAAACTTTCAGGTGTGT	22	2627
*miR-278-1*	TCGGTGGGACTTTCGTTCGT	20	2508
*miR-279-2*	GGGCGAGTTTGCTTCTGGTTC	21	1505
*miR-204-1*	TTCCCTTTGTCATCCTTCGCCT	22	1058
*miR-306-3*	TCAGGTACTAGGTGACTCTGAG	22	592
*miR-279-3*	TGACTAGATCTACACTCATTGA	22	467
*miR-282-3*	TAGCCTCTACTTGGCTTTGTCTG	23	464
*miR-31-1*	AGGCAAGAAGTCGGCATAG	19	408
*novel-miR-73*	CCGCCAAATCAGAAGTGCCCG	21	221,836
*novel-miR-75*	CCGCCAAATCAGAAGTGCCCG	21	221,834
*novel-miR-77*	CCGCCAAATCAGAAGTGCCCG	21	221,829
*novel-miR-40*	TCTTTGGTATCCTAGCTGTAGG	22	134,453
*novel-miR-245*	TGGAAGACTAGTGATTTTGTTGTTTT	26	18,495
*novel-miR-74*	GGCACTTCTGATTTGATGACT	21	12,983
*novel-miR-76*	GGCACTTCTGATTTGATGACT	21	12,980
*novel-miR-78*	GGCACTTCTGATTTGATGACT	21	12,980
*novel-miR-142*	TAGGAACTTCATACCGTGCTCTT	23	12,814
*novel-miR-79*	TCATAAGACACACGCGGCTCTCT	23	3049

* CPM formula: CPM = C/N × 1,000,000, where “C” represents the number of reads compared to this gene and “N” represents the total number of reads compared to the gene. The expression levels (CPM) were calculated as the average expression in antennae.

**Table 3:** Candidate miRNAs targeting chemosensory-related genes in *Loxostege sticticalis*.

miRNA	Sequence (5′-3′)	Target mRNA	RNAhybrid		miRanda	
			MFE *	*p*-Value	MFE *	Score
*let-7-4*	TGAGGTAGTAGGTTGTATGGTTT	*LstiOR8*	−27.2	0.040887	−25.4	165
*mi* *R-183-1*	TATGGCACTGGTAGAATTCACTGT	*LstiCSP5*	−30.2	0.004825	−27.2	158
*mi* *R-7911-1*	CTCCCGGCCGATGCACCA	*LstiOBP10*	−28.4	0.013131	−25.4	145
*mi* *R-2756-1*	CCCCTGGCTGCTACATCGTAT	*LstiOR3*	−32.7	0.003096	−26.8	171
*undef-* *miR-48*	CGGCGGCGGCGCGTGGCG	*LstiOBP4*	−32.9	0.002526	−26.8	162
*undef-* *miR-55*	ATCCCACCGCTGTCACCA	*LstiPBP2*	−29.8	0.003019	−25.3	176
*undef-* *miR-94*	TAGCAGCACGTAAATATTGGTG	*LstiGR63a.2*	−28.4	0.006389	−25.2	170
*undef-* *miR-158*	TGAGGTAGTTGGTTGTATGGT	*LstiGR21b*	−28.2	0.006776	−26.1	150
*undef-* *miR-316*	CCACTGCCCCAGGTGCTGCTGG	*LstiCSP10*	−37.9	0.001731	−35.9	149
*undef-* *miR-316*	CCACTGCCCCAGGTGCTGCTGG	*LstiOBP22*	−42.2	0.000035	−40.2	165
*undef-* *miR-321*	CTCCTGACTCCAGGTCCTGTG	*LstiCSP3*	−25.6	0.020271	−25.6	162
*undef-* *miR-353*	TCAGTGCATCACAGAACTTTGTA	*LstiOBP12*	−27.0	0.005103	−25.3	163
*undef-* *miR-398*	ACTGGACTTGGAGTCAGAAGG	*LstiGR63a*	−29.5	0.002488	−26.2	158
*undef-* *miR-460*	TGAGGGGCAGAGAGCGAGACTTT	*LstiOBP15*	−30.9	0.025787	−25.8	155
*undef-* *miR-521*	ACCCTGTAGCTGCTTAGGGGCG	*LstiGR45*	−28.8	0.010805	−26.1	156
*undef-* *miR-523*	CCATCCTTCGACTCGACTGGCG	*LstiIR7g*	−27.8	0.038807	−27.5	170
*novel-miR-7*	GTTCCGGTAGTATGCCCCTA	*LstiOBP17*	−28.9	0.004842	−25.0	160
*novel-miR-30*	TCACCATCGCTCGGCTGTCGCT	*LstiOBP26*	−35.5	0.000704	−29.8	168
*novel-miR-30*	TCACCATCGCTCGGCTGTCGCT	*LstiOR48*	−31.7	0.003536	−30.6	166
*novel-miR-31*	GTCGCCATCGCCATCGCTCG	*LstiOBP29*	−28.7	0.016774	−26.9	153
*novel-miR-103*	CGCGGCCGAGGGCGGCGCGGA	*LstiOR43*	−33.4	0.047515	−26.2	153
*novel-miR-132*	CTCGTCGTCGGCGCCGGCTCCG	*LstiOBP13*	−31.7	0.03937	−30.6	155
*novel-miR-137*	ATGGCAGTCGCGACTTTGCAAAT	*LstiGR5b*	−28.4	0.013829	−25.0	165

* MFE, minimum free energy.

## Data Availability

The raw data supporting the conclusions of this study will be provided to the reader upon request.

## Supplementary Materials

The following supporting information can be downloaded at: https://www.mdpi.com/article/10.3390/life14121705/s1, Figure S1: The length distribution of the reads of small RNA in the antennae of *Loxostege sticticalis*; Figure S2: The categories of small RNAs in male and female antennae of *Loxostege sticticalis*; Figure S3: The heatmap of Pearson correlation coefficients among the six constructed libraries of small RNAs in the antennae of *Loxostege sticticalis*; Figure S4: A bubble diagram of the GO function analysis and KEGG pathway enrichment of differentially expressed miRNAs (DEmiRNAs); Table S1: Information on primers used in qRT-PCR; Table S2: The abundant known and novel miRNAs in male and female antennae of *Loxostege sticticalis*; Table S3: Conserved microRNA (miRNA) families identified in antennae of *Loxostege sticticalis*; Table S4: Expression analysis of miRNAs (DEmiRNAs) in antennae of *Loxostege sticticalis*; Table S5: GO functional analysis of DEmiRNAs in antennae of *Loxostege sticticalis*; Table S6: KEGG pathway enrichment analysis of DEmiRNAs in antennae of *Loxostege sticticalis*.
