# Supplementary material for: Identification of miRNAs Involved in Olfactory Regulation in Antennae of Beet Webworm, Loxostege sticticalis (Lepidoptera: Pyralidae)
Source: Life (Basel). 2024 Dec 23;14(12):1705. doi: 10.3390/life14121705 (PMC11677245; doi:10.3390/life14121705)
Supplement: Supplementary file 1 [file life-14-01705-s001.zip › life-3322391-supplementary/Supplementary Figures.pdf]

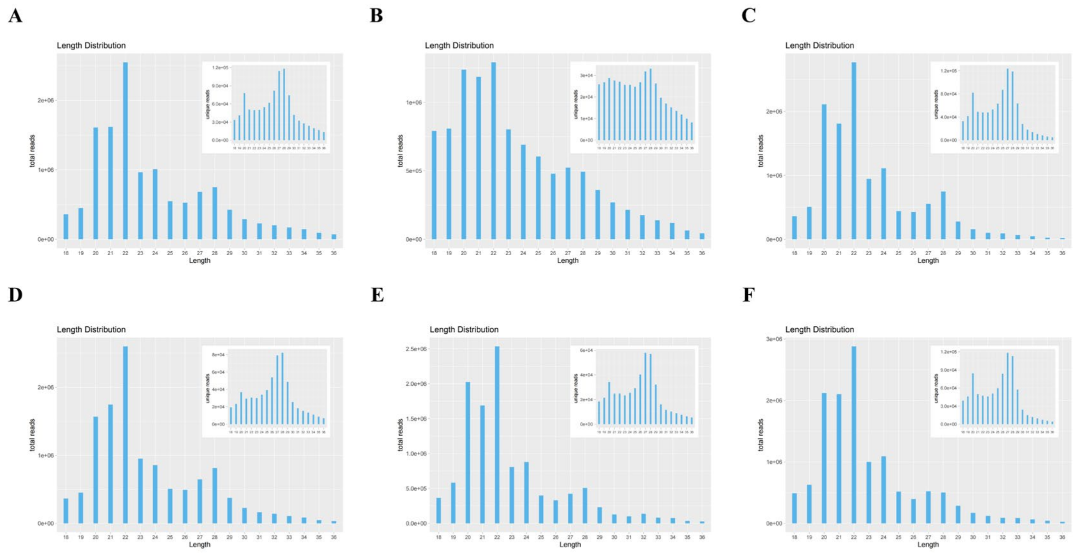

Figure S1. The length distribution of small RNA reads in the antennae of *Loxostege sticticalis*. The lower left corner showed that the length distribution of total reads, and the upper right corner showed that the length distribution of the unique reads. (**A-F)** represent the length distribution of three males (LstiMA1-3) and females (LstiFA1-3) antennae of *L. sticticalis*, respectively. The number (1-3) indicates biological replicates.

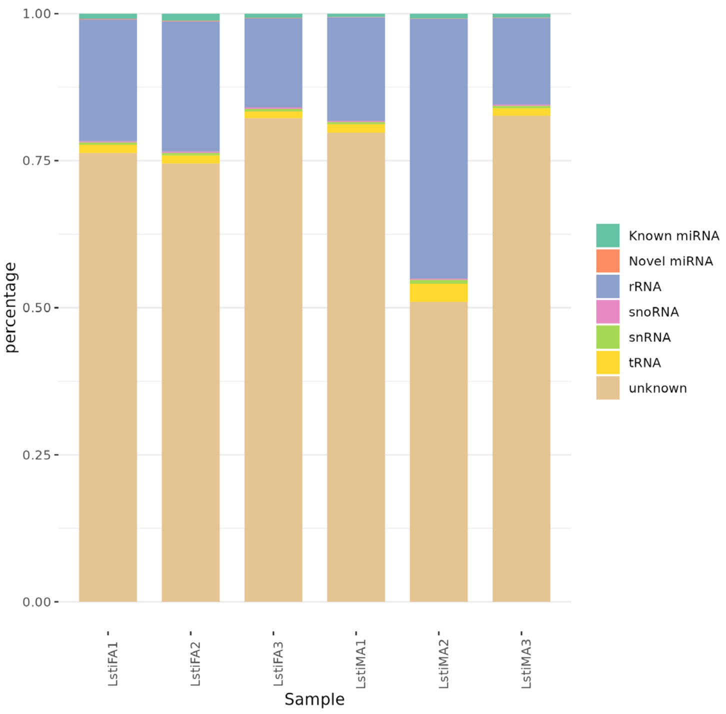

Figure S2. The categories of small RNA in male and female antennae of *Loxostege sticticalis*. FA represent female antennae, and MA represent male antennae. The number (1-3) indicates

biological replicates. rRNA, ribosomal RNA; tRNA, transfer RNA; snRNA, small nuclear RNA; snoRNA, small nucleolar RNA.

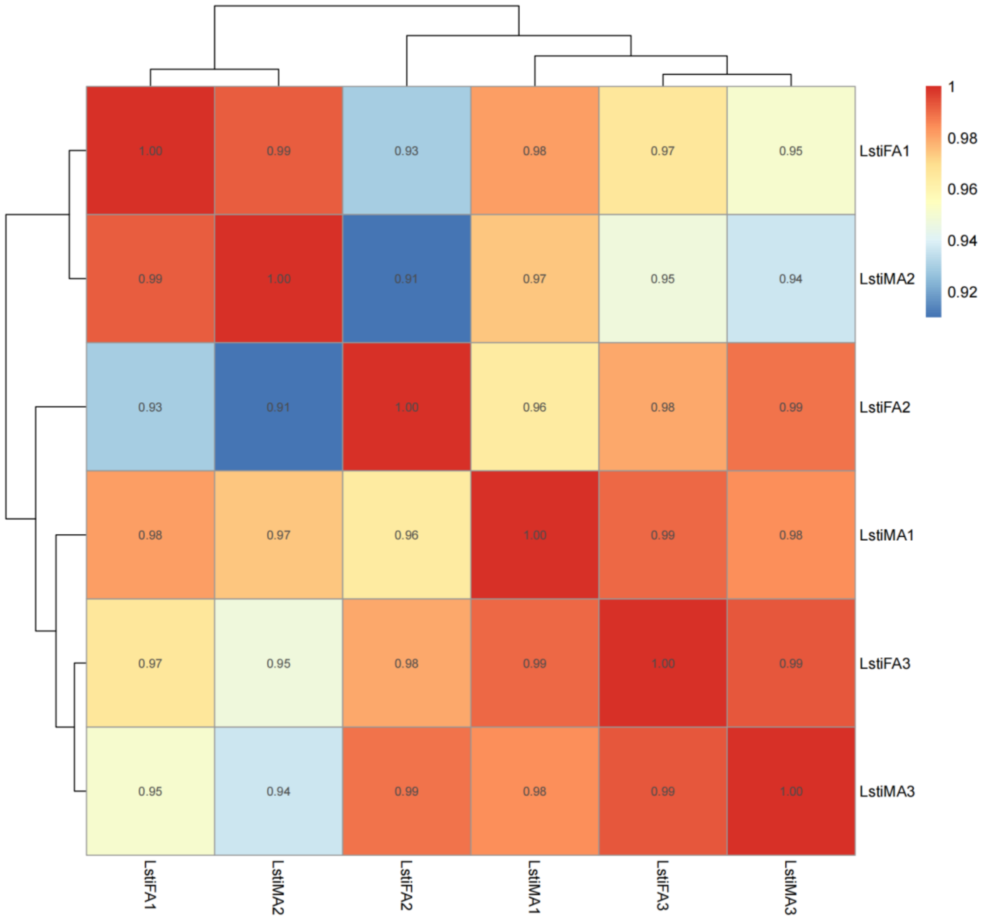

Figure S3. Heatmap of Pearson correlation coefficient among six constructed small RNAs libraries from the antennae of *Loxostege sticticalis*. The left and upper side are the clustering of the libraries, and the right and lower side are the names of the samples (Lsti, *Loxostege sticticalis*; MA, male antennae; FA, female antennae). The number (1-3) after each sample represent biological replicate. The squares with different colors represent the correlation of the two libraries.

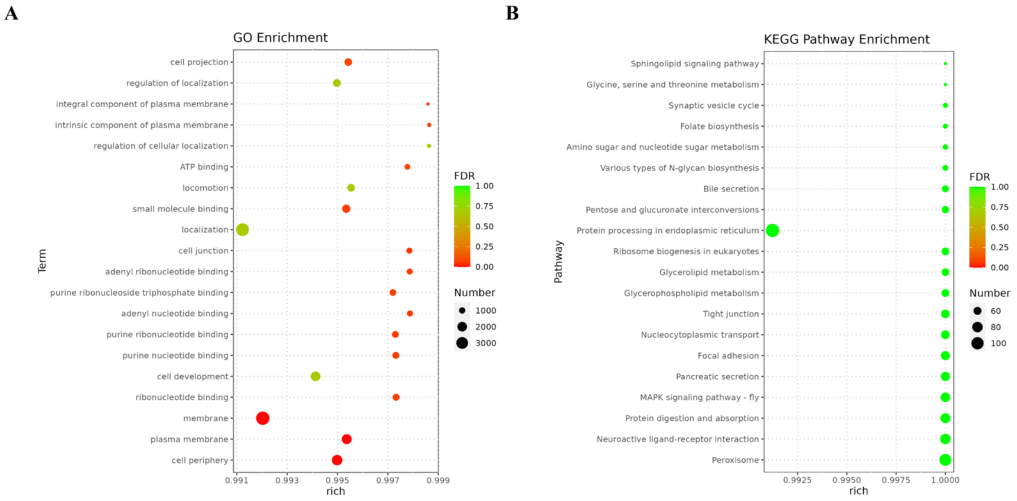

Figure S4 Bubble diagram of GO function analysis and KEGG pathway enrichment of differently expressed miRNAs (DEmiRNAs). (**A**) Top 20 GO enrichment terms of DEmiRNAs. (**B**) Top 20 KEGG pathways enrichment of DEmiRNAs.
